# *Acanthamoeba* Protease Activity Promotes Allergic Airway Inflammation via Protease-Activated Receptor 2

**DOI:** 10.1371/journal.pone.0092726

**Published:** 2014-03-21

**Authors:** Mi Kyung Park, Min Kyoung Cho, Shin Ae Kang, Hye-Kyung Park, Dong-Hee Kim, Hak Sun Yu

**Affiliations:** 1 Department of Parasitology, School of Medicine, Pusan National University, Yangsan, Republic of Korea; 2 Immunoregulatory therapeutics group in Brain Busan 21 project, Busan, Republic of Korea; 3 Department of Internal Medicine, School of Medicine, Pusan National University, Yangsan, Republic of Korea; 4 Department of Nursing, College of Nursing, Pusan National University, Yangsan, Republic of Korea; Baylor Institute for Immunology Research, United States of America

## Abstract

*Acanthamoeba* is a free-living amoeba commonly present in the environment and often found in human airway cavities. *Acanthamoeba* possesses strong proteases that can elicit allergic airway inflammation. To our knowledge, the aeroallergenicity of *Acanthamoeba* has not been reported. We repeatedly inoculated mice with *Acanthamoeba* trophozoites or excretory-secretory (ES) proteins intra-nasally and evaluated symptoms and airway immune responses. *Acanthamoeba* trophozoites or ES proteins elicited immune responses in mice that resembled allergic airway inflammation. ES proteins had strong protease activity and activated the expression of several chemokine genes (*CCL11*, *CCL17*, *CCL22*, *TSLP*, and *IL-25*) in mouse lung epithelial cells. The serine protease inhibitor phenyl-methane-sulfonyl fluoride (PMSF) inhibited ES protein activity. ES proteins also stimulated dendritic cells and enhanced the differentiation of naive T cells into IL-4-secreting T cells. After repeated inoculation of the protease-activated receptor 2 knockout mouse with ES proteins, airway inflammation and Th2 immune responses were markedly reduced, but not to basal levels. Furthermore, asthma patients had higher *Acanthamoeba*-specific IgE titers than healthy controls and we found *Acanthamoeba* specific antigen from house dust in typical living room. Our findings suggest that *Acanthamoeba* elicits allergic airway symptoms in mice via a protease allergen. In addition, it is possible that *Acanthamoeba* may be one of the triggers human airway allergic disease.

## Introduction

An airway allergic reaction is characterized by the synthesis of allergen-specific immunoglobulin (Ig) E and Th2 cytokines, such as interleukin (IL)-4, IL-5, and IL-13, which lead to the recruitment and sensitization of effector cells such as eosinophils, basophils, and mast cells [1]. Antigen recognition and uptake by innate immune cells is the first step in the process of antigen presentation, which can lead to the initiation of the adaptive immune response [2]. The allergic cascade starts with the recognition of allergens by antigen presenting cells, mainly dendritic cells (DCs); leads to Th2 polarization and a switch to IgE production by B cells; and culminates in mast cell sensitization and triggering [2]. Antigens are recognized by a diverse set of pattern recognition receptors, such as Toll-like receptors, C-type lectin receptors, and protease-activated receptor (PAR) 2 on immune or non-immune cells [2], [3]. Allergic reactions are induced by various environmental allergens. Many allergens exhibit intrinsic protease activities, and some proteases from infectious agents, parasites, and fungi have been identified as potent allergens [4]–[8]. These protease allergens induce Th2 immune reactions by activating several chemokines and cytokines [8]. Thus, protease activities may be critical to the initiation of allergic responses.

Recently, some proteolytic allergens were shown to cause the breakdown of epithelial barriers through mechanisms mediated by PAR2. Activation of PAR promotes cytokine production and leukocyte activation [9], [10]. PARs belong to the recently described family of G protein-coupled seven transmembrane domain receptors [11], [12]. PARs are activated via the proteolytic cleavage of their N-terminal domain by proteinases. Cleavage generates a new N-terminal “tethered ligand,” which can autoactivate receptor activity [13]. Four members of the PAR family have been cloned thus far. PAR1, PAR3, and PAR4 are activated by thrombin, and PAR2 is activated by serine proteases, including trypsin, tissue kallikreins, coagulation factors VIIa and Xa, mast cell tryptase, and transmembrane serine proteases [14]. PAR1 and PAR2 are widely distributed in the airway, including within human nasal primary epithelial cells. PAR2 acts primarily as a pro-inflammatory molecule in the respiratory system, and it can be activated by exogenous proteinase allergens [15].

We introduced the possibility of free-living amoebae (FLA) as new aeroallergens. One FLA, *Acanthamoeba*, is an opportunistic protozoan widely distributed in the environment. *Acanthamoeba* can cause serious human infections, including blinding keratitis and fatal encephalitis [16]. *Acanthamoeba* species have been isolated from public water supplies, swimming pools, air-conditioning units (cooling towers), sewage, compost, sediments, soil, beaches, vegetables, surgical instruments, contact lenses and their cases, and the atmosphere [16]. In addition, *Acanthamoeba* species have been recovered from human nasal cavities, pharyngeal swabs, lungs tissues, and skin lesions [16]–[18]. Unsurprisingly, anti-*Acanthamoeba* antibodies have been identified in the majority of healthy individuals tested, indicating that exposure to FLA is common [19]. In addition, many proteases, including serine and cysteine proteases, have been isolated from *Acanthamoeba* [20]–[23]. A central role for these proteases in diverse processes, such as differentiation, host cell invasion and egress, catabolism of host proteins, cyto-adherence, and stimulation and evasion of host immune responses, has been proposed [23]–[25]. However, despite the ubiquitous presence of *Acanthamoeba* in the environment and its possession of proteases strong enough to elicit allergic airway inflammation, to our knowledge, no report on the role *Acanthamoeba* in allergic airway inflammation has been published to date.

In this study, we repeatedly inoculated mice intranasally with *Acanthamoeba* trophozoites or excretory-secretory (ES) proteins and evaluated symptoms and immune responses. In addition, we investigated the role of DCs and PAR2 in airway inflammation induced by *Acanthamoeba*.

## Materials and Methods

### *Acanthamoeba* cultivation and ES protein preparation

*Acanthamoeba lugdunensis* KA/WP6 strain, isolated from domestic tap water in Korea, was maintained in PYG medium [26]. The KA/WP6 strain has the same molecular characteristics as the *A. lugdunensis* L3A strain (ATCC 50240) [26]. To obtain ES proteins, live trophozoites were incubated in PYG medium for one week at 25°C. Following centrifugation at 12,000×*g* for 30 min, supernatants were concentrated using 3000-Da centrifugal filter units (Millipore Co., Billerica, Massachusetts, USA). HiTrap Desalting™ (GE Healthcare, Little Chalfont, United Kingdom) was performed to eliminate excess salts from the collected medium. The samples were then dialyzed against PBS. After obtaining ES proteins, the ToxinSensor Gel Clot Endotoxin Assay Kit (GenScript, Piscataway, New Jersey, USA) was used to eliminate endotoxins.

### Animals and experimental design

Female C57BL/6 mice (six weeks old) were purchased from Samtako Co. (Gyeonggi-do, Korea). PAR2^−/−^ mice (C57BL/6 background) were purchased from The Jackson Laboratory (Bar Harbor, Maine, USA) and were bred in a specific pathogen-free facility at the Institute for Laboratory Animals of Pusan National University. Mice in the infected group were administered 100 (low dose) or 5×10^4^ (high dose) *A. lugdunensis* trophozoites intranasally on days 0, 2, 4, 6, 8, and 10. Using the same protocol, mice in the other groups were treated with 20 μg of ES proteins or PMSF pre-treated ES proteins under light anesthesia using Zoletil 50 (Virbac Laboratories, Courbevoie Cedex, France). On the day after the last infection, airway hypersensitivity reactions were measured, and animals were sacrificed. Serum was collected by cardiac puncture, and lung-draining lymph nodes (LLNs) were excised. Lymphocytes were isolated from LLNs as previously described [27]. Briefly, LLNs were disrupted and treated with ammonium-chloride-potassium (ACK) hypotonic lysis solution to lyse red blood cells. The remaining cells were filtered through a 100-μm mesh and plated in 48-well plates. For CD3 stimulation experiments, 0.5 μg/ml of CD3 antibody was added to the plated cells. These cells were incubated for 72 hr at 37°C in a 5% CO_2_ atmosphere. Following incubation, the culture medium were harvested and stored at −20°C for ELISA.

### Analysis of bronchoalveolar lavage fluid (BALF) and differential cell counting

To obtain BALF, the tracheas of anesthetized mice were exposed and cut just below the larynx. A polyurethane flexible tube (outer diameter: 0.4 mm, length: 4 cm; BD Biosciences, San Jose, California, USA) attached to a blunt 24-gauge needle was placed in the trachea, and the lung was lavaged once with 800 μl of sterile, warm PBS. BALF samples were centrifuged for 5 min at 1500 rpm at 4°C. Supernatants were then decanted and immediately frozen at −70°C. Cell pellets were resuspended and washed twice in PBS. Total cell numbers were counted using a hemocytometer. To determine the differential cells counts, BALF cell smears were prepared with a cytospin apparatus and stained with Diff-Quik solution (Sysmex Co., Kobe, Japan), in accordance with conventional morphological criteria. At least 500 cells per slide were evaluated to obtain differential leukocyte counts.

### ELISA

ELISA was used to determine the levels of total IgE and *Acanthamoeba*-specific IgE in sera and the levels of IL-4, IL-5, IL-13, IFN-γ and IL-17A in BALF, the culture supernatants of lymphocytes from LLNs, and the supernatants of naive T cells co-cultured with BMDCs, according to the manufacturer's instructions (eBioscience, San Diego, California, USA). To estimate the level of *Acanthamoeba*-specific IgE, 96-well plates (Nunc, Roskilde, Denmark) were coated with ES proteins (final concentration: 1 μg/ml) in 0.1 M sodium carbonate buffer, pH 9.6, at 37°C until dry. The absorbance was measured at 450 nm using an ELISA plate reader. To eliminate the possibility of cross-reactions between *Acanthamoeba* and known allergens, the sera were reacted with Der P1 and *Aspergillus* protease for 30 min before ELISA.

### Lung histopathology

Histological analyses were conducted as described previously [28]. In brief, lung tissues were fixed with formaldehyde and embedded in paraffin. Thin sections of the embedded tissues were then stained with hematoxylin and eosin (H&E) and periodic acid-Schiff (PAS) and examined microscopically.

### Zymography for detection of protease activity

A previously described method was used for zymography analysis [23], [29]. Briefly, a gel containing 0.1% gelatin was prepared. ES proteins treated with or without protease inhibitors were loaded onto the gelatin gel and separated by electrophoresis with 1× Tris-glycine SDS running buffer (0.025 M Tris, 0.192 M glycine, pH 8.5, 0.1% SDS) at 125 V for 2 h. The gels were rinsed several times, incubated in renaturing buffer, and then stained with Coomassie brilliant blue, as described previously [29]. Clear bands against a stained background indicated regions of gelatin digestion by proteases.

### Analysis of Th2 related gene expression in lung epithelial cells

Mouse lung epithelial cells (MLE12 cells) were obtained from ATCC (Manassas, VA, USA). Real-time PCR was performed using an iCycler™ (Bio-Rad, Hercules, California, USA) to determine the levels of CCL17 (thymus and activation-regulated chemokine, TARC), CCL22 (macrophage-derived chemokine, MDC), CCL11 (eotaxin), thymic stromal lymphopoietin (TSLP), and IL-25 mRNA. GAPDH was used as an internal reference. Primers and PCR conditions have been described previously [30]–[32].

### Generation of bone marrow-derived DCs (BMDCs)

BMDCs were differentiated from bone marrow cells by methods previously reported, with modification [33], [34]. In brief, bone marrow cells were flushed from the femurs and tibias of 7-week-old C57BL/6 mice, washed, and cultured in complete RPMI 1640 medium containing 10% heat-inactivated fetal bovine serum (FBS), 50 μM 2-mercaptoethanol, 2 mM glutamine, penicillin and streptomycin (100 U/ml and 100 μg/ml, respectively; Invitrogen, Carlsbad, California, USA), and recombinant mouse granulocyte macrophage colony-stimulating factor (GMC-SF) and recombinant mouse IL-4 (10 ng/ml each; R&D Systems, Minneapolis, Minnesota, USA). Non-adherent granulocytes were removed after 24 hr of culture, and fresh complete medium was added every other day. All cultures were incubated at 37°C in 5% CO_2_. After 7 days of culture, >80% of the cells expressed characteristic DC-specific markers (CD11c^+^) as determined by flow cytometry.

### *In vitro* BMDC stimulation assay

Immature BMDCs were cultivated in 6-well plates at 5×10^6^ cells/well in 3 ml of complete RPMI 1640 medium enriched with 10% FBS and 40 ng/ml GM-CSF. BMDCs were stimulated with 1 μg/ml of LPS or 5 μg/ml of ES proteins for 48 hr. BMDCs stimulated with ES proteins were then incubated with the following anti-mouse antibodies: FITC-conjugated anti-CD11c and PE-conjugated anti-MHCII, -CD40, -CD80 and -CD86 (all from eBioscience). Flow cytometry was performed using a FACS Canto II cytometer (BD Biosciences) equipped with Canto software.

### Naive T-cell differentiation and cytokine production

After BMDCs were stimulated with ES proteins or LPS, they were incubated with naive T cells. Th2 differentiation was analyzed by FACS and ELISA. BMDCs (100 μl) were seeded in round-bottom wells of 96-well culture plates at 2×10^5^ cells/well and were stimulated with 5 μg/ml ES proteins or 1 μg/ml LPS for 48 hr at 37°C. Naive T cells were isolated from the spleens and lymph nodes of C57BL/6 mice using a CD4^+^CD62L^+^ T-cell isolation kit (Miltenyi Biotec, Bergisch Gladbach, Germany). The isolation of naive T cells was a two-step process. First, non-CD4^+^ T cells were depleted in a negative selection step by incubating cells with a cocktail of biotin-conjugated antibodies and anti-biotin microbeads. Second, CD4^+^CD62L^+^ T cells were labeled with CD62L microbeads and isolated by positive selection. Isolated naive T cells were co-cultured with BMDCs at a BMDC:T cell ratio of 1∶5 for 72 hr at 37°C. Subsequently, for CD3 stimulation experiments, 0.5 μg/ml of CD3 antibody was added to the cells. Cells were incubated for 72 hr at 37°C. T cells were then incubated with GolgiPlug (BD Biosciences), stained with FITC-labeled anti-CD4 (eBioscience) in accordance with the manufacturer's recommended protocol, fixed, permeabilized, and stained intracellularly with PE-Cy7-labeled anti-mouse IL-4. The Th2 differentiation rate was analyzed by flow cytometry. Total RNA isolation and cDNA synthesis from co-cultured naïve T cells and perform real-time PCR to determine expression levels of T-bet, GATA-3, ROR-γ and Foxp3. GAPDH levels were used to normalize RNA content of samples.

### Evaluation of *Acanthamoeba*-specific IgE levels in asthma patients

To investigate the *Acanthamoeba*-specific IgE levels in asthma patients, 25 patients diagnosed with asthma and 9 non-atopic healthy controls were recruited to the study from January to December 2010. Asthma was diagnosed based on history and positive airway hyperresponsiveness to methacholine challenge. Airway hyperresponsiveness was expressed as the drug concentration required to cause a 20% decrease in forced expiratory volume (FEV1) (PC20) in non-cumulative units, as determined by methacholine challenge testing, and was regarded as positive if the PC20 was <8 mg/ml. The level of *Acanthamoeba*-specific IgE in the serum was measured using ELISA inhibition tests, in accordance with the manufacturer's instructions (eBioscience). All assays were performed in duplicate. The cut-off value for detecting *Acanthamoeba*-specific IgE levels in serum was determined from the mean plus the two-fold SD of the absorbance values from non-atopic healthy controls. Competitive ELISA inhibition tests were performed to determine the allergenic cross-reactivity between *Acanthamoeba* and known allergens.

### Detection of *Acanthamoeba*-derived antigen in house dust

House dust was collected from a bed mattress and carpet in an apartment using a vacuum (vacuum pore size: 0.34 mm). The house dust was dissolved in filtered PBS and mixed by vortexing and shaking for 1 hr. The house dust solution was stored at −70°C. *Acanthamoeba*-specific antigen from the house dust was detected by ELISA. A 96-well plate (Nunc) was coated at 37°C until dry with 5 μg/ml of house dust solution. After 3 washes with PBS containing 0.1% Tween 20 (Sigma), diluted serum (1∶500) from *Acanthamoeba*-infected mice was added to the plate and incubated for 2 hr at 37°C. After incubation, the *Acanthamoeba*-specific IgG1 level was estimated by ELISA.

### Statistical analysis

All experiments were performed in triplicate. Student's *t*-test or ANOVA was used to calculate the mean ± SD and to determine significant differences. Statistical analysis was performed with PASW 18.0.

### Ethics Statement

All animal studies were approved by the Pusan National University Animal Care and Use Committee (Approval No. PNU-2010-000165). Human serum specimen was provided by national biobank of Korea, PNUH. Written informed consent was obtained from each participant before enrollment. Participation was entirely voluntary. Participants were free to refuse to participate or withdraw from the study at any time, and were informed that only the aggregate data would be reported. Participants were also told that there would be no disadvantages for canceling their participation. The study protocol using the sample was approved by the Ethics Committee of Pusan National University Hospital (IRB Approval No. PNUH-2010-5). We used the same dust samples of previous study [35]. Dust was collected from private apartment houses after getting permission from people who lived in each house.

## Results

### Treatment with *Acanthamoeba* trophozoites elicits strong allergic airway inflammation

**Figure 1:**
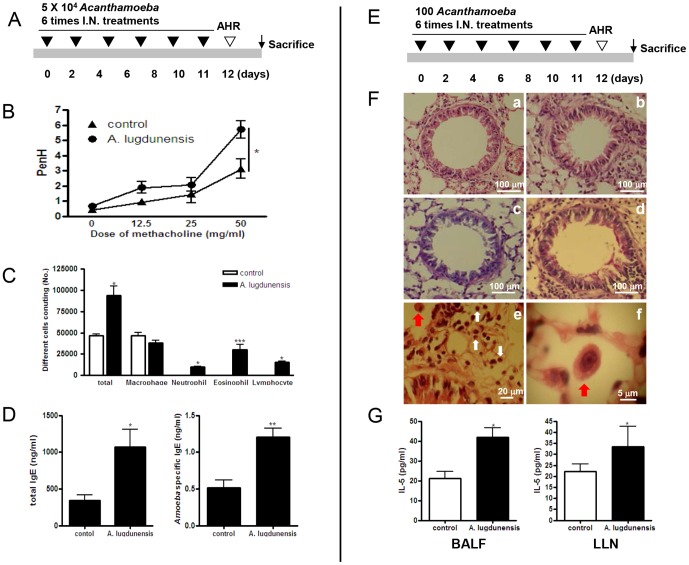
*Acanthamoeba* trophozoites induce allergic airway inflammation. Allergic airway inflammation was induced by inoculation of mice with high (A–D) and low (E–G) doses of *Acanthamoeba*. (A) Intranasal inoculation schedule for the high-dose (4×10^5^ *Acanthamoeba* trophozoites) model. (B) Airway resistance values in response to methacholine (0 to 50 mg/ml). (C) Differential cell count in 800 μl BAL after Diff-Quik staining. (D) Total and *Acanthamoeba*-specific IgE levels were measured in serum by ELISA. (E) Intranasal inoculation schedule for the low-dose (100 *Acanthamoeba* trophozoites) model. (F) Tissue inflammation observed on stained lung sections (a and c: PBS-treated; b and d: *Acanthamoeba*-infected; a and b: H&E-stained; c and d: PAS-stained; red arrows indicate *Acanthamoeba* trophozoites and white arrows indicate eosinophils). (G) Cytokine concentrations in BALF and in the culture medium of CD3-stimulated lymphocytes isolated from LLNs. (*p<0.05, **p<0.01, ***p<0.001; n = 7, three independent experiments).

To demonstrate the effects of *Acanthamoeba* on allergic airway inflammation, we introduced *Acanthamoeba* trophozoites (5×10^4^) into the airways of mice and assessed biological and pathological changes (Figure 1A). Dose-dependent increases in airway hyperresponsiveness to methacholine were observed in mice inoculated with *Acanthamoeba* trophozoites (Figure 1B). Inflammatory cell infiltration was also observed in the airways, and the numbers of eosinophils, neutrophils, and lymphocytes increased in BALF (Figure 1C). Histological examination of the lungs in the *Acanthamoeba*-treated group revealed massive inflammatory cell infiltration, bronchial epithelial cell hyperplasia, and goblet cell hyperplasia (Figure S1A). In addition, the levels of IL-4, -5, and -13 (Th2 cytokines) in BALF and in the culture medium of LLN T cells increased in the *Acanthamoeba*-infected group, compared with levels in the control group (Figure S1B). For *Acanthamoeba*-infected mice, the amount of IFN-γ level in LLNs increased, but IL-17 levels in BALF and LLNs did not change. Finally, marked increases in total serum IgE and *Acanthamoeba*-specific IgE were observed after *Acanthamoeba* infection (Figure 1D).

To know the general infection dose of *Acanthamoeba* in nature system, we investigated previous reports about the average number of infected *Acanthamoeba* trophozoites in several environment sources. We determined infection dose of *Acanthamoeba* according to average number of *Acanthamoeba* trophozoites in 1 L contaminated domestic tap water [36]. Occasionally, we could find 1000 over *Acanthmaoeba* trophozoites in 1 L tap water. It might be possible that 100 *Acanthamoeba* trophozoites contact to normal person at one time who used *Acanthamoeba* contaminated tap water. To determine whether a low dose of *Acanthamoeba* elicited allergic airway inflammation, we intranasally administered 100 *A. lugdunensis* trophozoites into the airways of mice six times (Figure 1E). Histological examination showed mild infiltration of inflammatory cells into the lungs and hyperplasia of some bronchial epithelial cells after treatment with *A. lugdunensis* (Figure 1F-b). In addition, mucin production by goblet cells was slightly higher in infected mice than in control mice (Figure 1F-d). Some *Acanthamoeba* were observed in alveoli, and several eosinophils were detected around *Acanthamoeba* (Figure 1F-e and -f). PenH values slightly increased after methacholine administration in the low dose *Acanthamoeba* treated group, but it is not significant (Figure S2A). In airways, the proportion of immune cells increased slightly after *Acanthamoeba* administration; the number of eosinophils increased considerably (Figure S2B). In BALF and LLNs, IL-5 levels increased after *Acanthamoeba* administration (Figure 1G); however, IL-4 and IL-13 levels did not change (data not shown). Total serum IgE and *Acanthamoeba*-specific IgE did not increase after low-dose *Acanthamoeba* infection (Figure S2C).

### The protease activity of ES proteins induces Th2 chemokine production in lung epithelial cells

**Figure 2:**
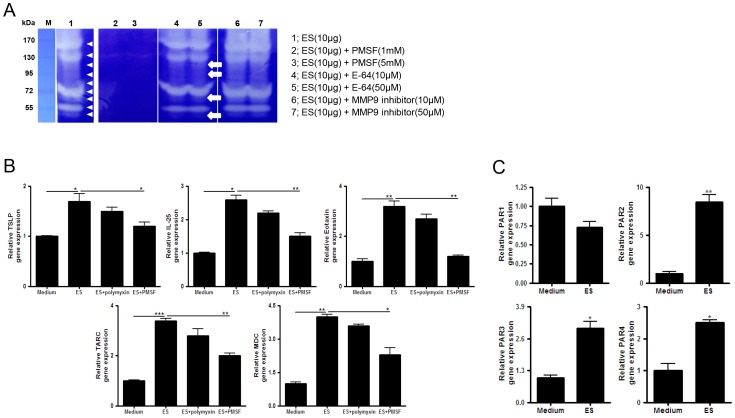
ES proteins elicit Th2-related chemokine production in the absence of protease inhibition. (A) ES proteins were treated with various protease inhibitors. The samples were incubated for 2 hr and were assayed by zymography on 0.1% gelatin SDS-PAGE gels (lane 1: 10 μg ES proteins; lane 2: with 1 mM PMSF; lane 3: with 5 mM PMSF; lane 4: with 10 μM E-64; lane 5: with 50 μM E-64; lane 6: with 10 μM MMP-9 inhibitor; lane 7: with 50 μM MMP-9 inhibitor; arrowhead: protease activity of ES proteins; arrow: protease activity inhibited by E64). (B) Th2-related chemokine gene expression (*TSLP*, *TARC*, *MDC*, eotaxin, and *IL-25*) was measured in MLE12 cells after incubation with 1 μg/ml of ES proteins (ES) for 2 hr or pre-treatment with 0.1 mM PMSF and 10 μg/ml polymyxin B (polymyxin) for 2 hr. (C) The fold-change in PAR mRNA levels in MLE12 cells treated with ES proteins relative to those treated with medium, as detected by real-time RT-PCR. (*p<0.05, **p<0.01, ***p<0.001; three independent experiments).

To evaluate the protease activities of ES proteins, we used zymogram analysis with several types of protease inhibitors. Several proteases in the ES protein population digested gelatin. Their activities were completely inhibited by the serine protease inhibitor PMSF. The strongest activity band was not affected by E-64 (cysteine protease inhibitor) treatment, but some weaker activity bands disappeared. The matrix metallopeptidase (MMP)-9 inhibitor did not inhibit the activity of most proteases (Figure 2A). Treatment with ES proteins increased *TSLP*, *TARC*, *MDC*, *eotaxin*, and *IL-25* gene expression (known to be critical for the initiation and expansion of the Th2 response in lung epithelial cells) in MLE12 cells. The increase in gene expression was not inhibited by polymyxin B treatment but was significantly inhibited by serine protease inhibitors (Figure 2B). Treatment with ES proteins also increased PAR2, PAR3, and PAR4 mRNA levels in MLE12 cells. In particular, *PAR2* expression was more than 8-fold higher in ES-treated cells than in control cells (Figure 2C).

### ES proteins accelerate the differentiation of Th2 cells into CD4^+^T cells by activating DCs

**Figure 3:**
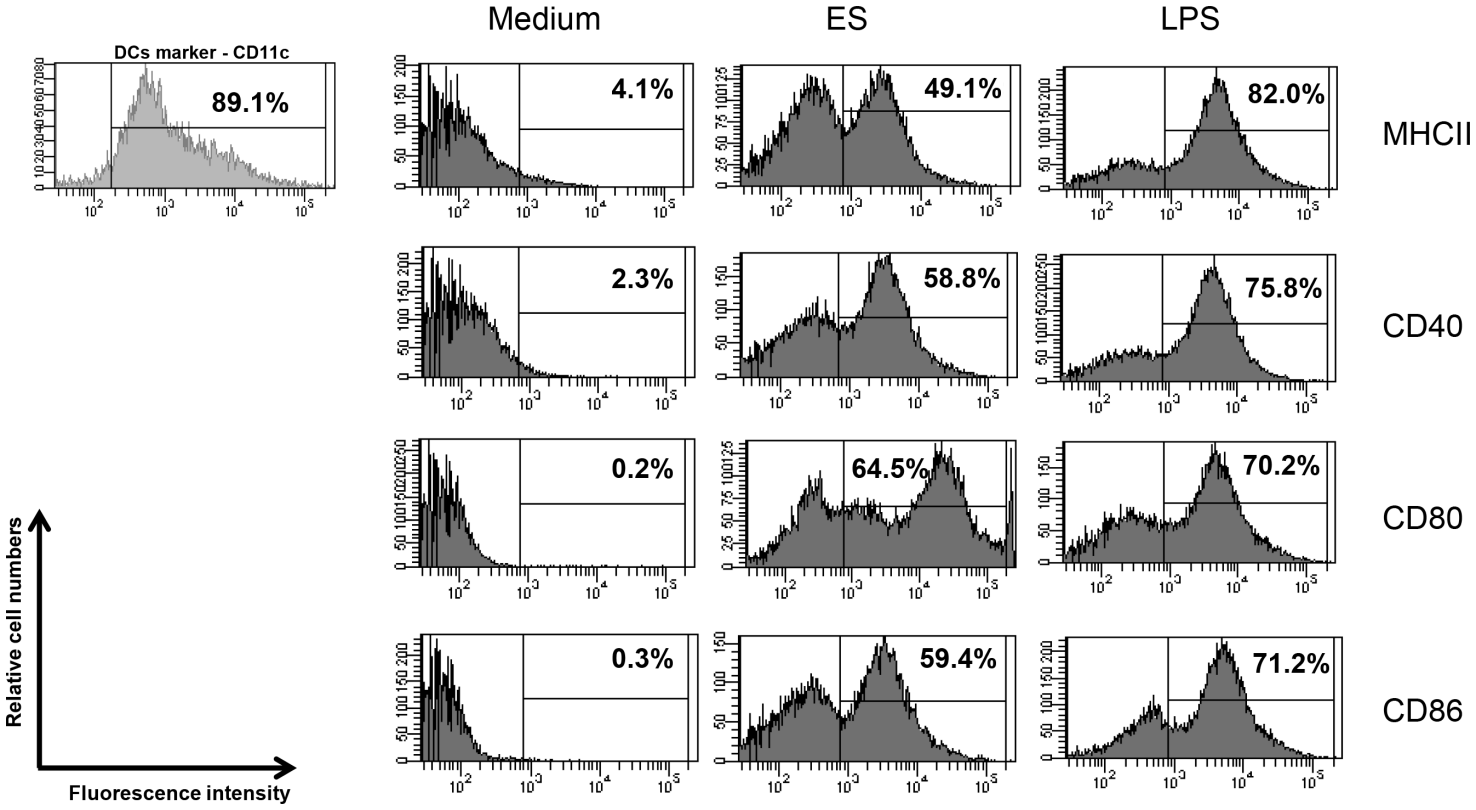
Activation of BMDCs by ES proteins. Expression of cell surface markers (MHCII, CD40, CD80, and CD86) on mouse BMDCs pulsed with ES proteins or LPS for 48 hr, compared with expression in untreated cells. (three independent experiments).

**Figure 4:**
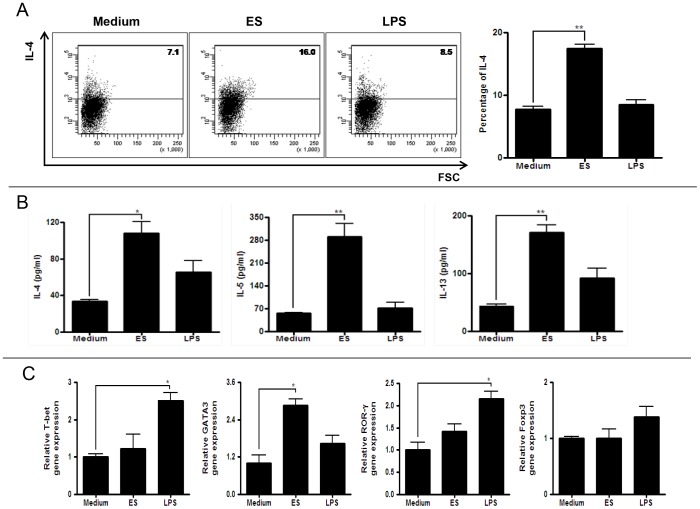
Differentiation of Th2 cells from naive T cells after co-cultivation with BMDCs activated by ES proteins. (A) Naive T cells were cultured with stimulated BMDCs by ES proteins or LPS or non-stimulated BMDCs for 3 days in the presence of anti-CD3 antibody. After gated with CD4^+^ T cells, IL-4 producing T cells were counted. (Medium: naive T cell with non-stimulated BMDCs; ES: naive T cell with BMDCs stimulated with ES proteins; LPS: naive T cell with BMDCs stimulated with LPS). (B) Cytokine levels in the supernatants from naive T cell and BMDC co-cultures were measured by ELISA. (C) The gene level was evaluated by realtime-RT PCR from naive T cell and BMDC co cultures (*p<0.05, **p<0.01, ***p<0.001; three independent experiments).).

Activated DCs are characterized by increased expression of co-stimulatory molecules (e.g., CD80 and CD86), MHCII and CD40 on their surface [37]. We cultured DCs (89% cells expressed CD11c, maturation marker) in the presence or absence of ES proteins or LPS (positive control) and analyzed surface markers using flow cytometry. The results showed that ES proteins stimulated DCs: the expression of MHCII, CD40, CD80, and CD86 was higher than in DCs cultured in medium alone (Figure 3). When ES protein-activated DCs were co-cultured with naive T cells (CD4^+^CD25^−^CD62L^+^ T cells), the number of IL-4-secreting CD4^+^ T cells (Th2 cells) was higher than in the control group (medium-treated group) (Figure 4A). In addition, IL-4, IL-5, and IL-13 production by CD4^+^ T cells increased when the cells were co-cultured with ES protein-activated DCs (Figure 4B). Furthermore, co-cultured naïve T cells with ES proteins treated DCs highly expressed GATA-3 transcription factor. (Figure 4C).

Serine protease dependant manner ES proteins

### ES proteins induce severe allergic airway inflammation by serine protease dependant manner, it's effective in part via PAR2

**Figure 5:**
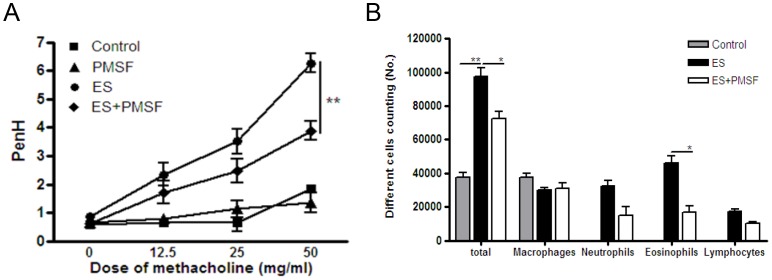
ES protein-induced allergic airway inflammation was inhibited by PMSF treatment. (A) Airway resistance values in response to methacholine (0 to 50 mg/ml). (B) Differential cell count in 800 μl BAL after Diff-Quik staining. (*p<0.05, **p<0.01, ***p<0.001; n = 15).

In order to know *in vivo* effects of serine protease inhibitor on ES protein induced inflammation, we treated ES protein with 20PMSF and evaluated airway inflammation. Most of inflammation index by ES protein treatment was significantly decreased by PMSF treatment. The airway hyper responsiveness to methacholine for ES protein were significantly decreased by treatment with PMSF (Figure 5A). Also, the infiltration of immune cells decreased after treated with PMSF, compared with only ES proteins treatment group; especially, the number of eosinophils decreased significantly (Figure 5B).

**Figure 6:**
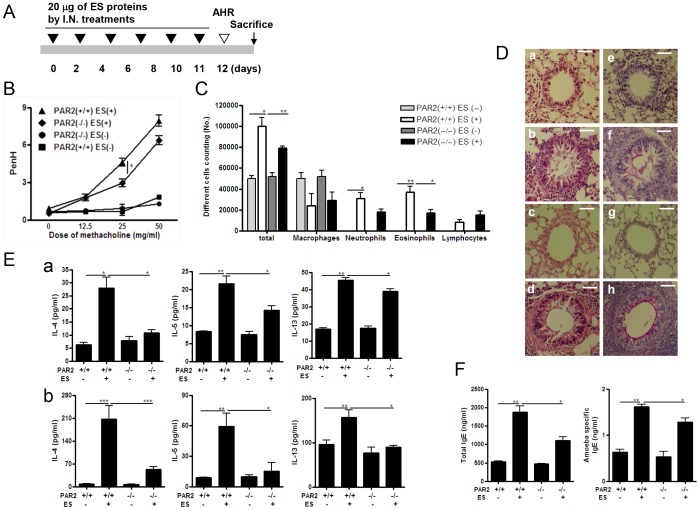
ES protein-induced allergic airway inflammation is reduced in PAR2 KO mice. (A) Intranasal inoculation schedule for the ES protein model. (B) Airway resistance values in response to methacholine (0 to 50 mg/ml). (C) Differential cell count in 800 μl BAL after Diff-Quik staining. (D) Tissue inflammation observed on stained lung sections (a and e: PBS-treated PAR2^+/+^ mice; b and f: ES protein-treated PAR2^+/+^ mice; c and g: PBS-treated PAR2^−/−^ mice; d and h; ES protein-treated PAR2^−/−^ mice; a, b, c and d: H&E-stained; e, f, g and h: PAS-stained). (E) Cytokine concentrations in BALF (a) and in the culture medium of CD3-stimulated lymphocytes isolated from LLNs (b) were measured. (F) Total and *Acanthamoeba*-specific IgE levels were measured in serum by ELISA. (*p<0.05, **p<0.01, ***p<0.001; n = 3∼5, three independent experiments).).

To determine whether PAR2 mediates the allergic airway inflammation induced by ES proteins of *Acanthamoeba*, we inoculated PAR2 knockout (KO) and wild-type (WT) mice with *Acanthamoeba* ES proteins six times (Figure 6A). For WT and PAR2 KO mice, PenH values increased after methacholine administration in the ES protein-treated group, compared with values in the control group (Figure 6B); however, responsiveness was slightly lower in the KO mice (Figure 6B). From WT and PAR2 KO mice, the numbers of inflammatory cells, particularly eosinophils and neutrophils, was significantly higher in the ES protein-treated group than in the control group (Figure 6C); however, the number of infiltrating eosinophils was lower in the KO mice (Figure 6C). The lungs of WT and KO mice administered ES proteins showed significant immune cell infiltration around the bronchial tracts, enhanced mucin production, and hyperplasia of lung epithelial cells and goblet cells (Figure 6D). The levels of IL-4, -5, and -13 in BALF and LLNs increased when mice were treated with ES proteins; however, the cytokine levels were lower in the PAR2 KO mice (Figure 6E). IL-17 and IFN-γ levels in BALF and LLNs were not affected by ES protein treatment in any of the experimental groups (data not shown). Finally, total serum IgE and *Acanthamoeba*-specific IgE levels were markedly higher when mice were treated with ES proteins, but amoeba-specific IgE levels in PAR2 KO mice were lower than in WT mice (Figure 6F).

### Asthma patients have a high level of *Acanthamoeba*-specific IgE

**Figure 7:**
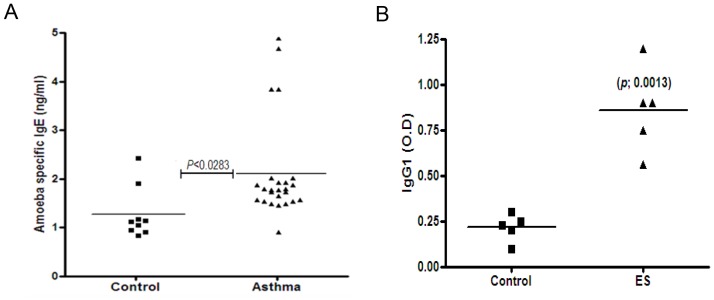
*Acanthamoeba*-specific IgE levels in asthma patients and *Acanthamoeba*-specific antigens in house dust. (A) After serum absorption with Der P1 and *Aspergillus* protease, the levels of *Acanthamoeba*-specific IgE in the sera of asthma patients were evaluated by ELISA. (B) Control: sera of 9 healthy individuals; Asthma: sera of 25 asthma patients. Immune-plates were coated with 5 μg/ml of house dust solution and then incubated with diluted serum (1:500) from *Acanthamoeba*-infected mice. Specific IgG1 levels were estimated by ELISA. (*p<0.05, **p<0.01, ***p<0.001).

To determine whether *Acanthamoeba* causes airway allergic reactions in humans, we evaluated *Acanthamoeba*-specific IgE levels in patients with asthma. The average IgE level in asthma patients was significantly higher (*p* = 0.028) than that in healthy controls (Figure 7A).

### *Acanthamoeba*-specific antigens are present in house dust

To assess whether ES proteins are present in domestic environments, we collected 5 house dust samples from typical living rooms. The house dust samples were incubated with serum from *Acanthamoeba*-infected mice or uninfected mice. After incubation with dust proteins, the IgG1 level in serum from *Acanthamoeba*-infected mice was higher than the IgG1 level in serum from of uninfected mice (Figure 7B).

## Discussion

The free-living amoeba *Acanthamoeba* is abundantly present in our environment. *Acanthamoeba* produces strong proteases, indicating that the amoeba might be a new allergenic agent. However, there are no reports to this effect. In this study, we investigated the ability of *Acanthamoeba* to elicit allergic airway inflammation *in vivo* and assessed whether *Acanthamoeba* culture products (ES proteins) induce a Th2 response. Principally, we found that *Acanthamoeba* and its culture products elicited allergic airway inflammation responses in experimental mice. ES proteins included many proteases, which seemed to have a central role in the Th2-mediated airway inflammation response *in vivo* and *in vitro*. ES proteins promoted DC maturation and activation, and the activated DCs promoted the differentiation of T cells into a Th2 cell population.

The proteases of *Acanthamoeba* have been previously demonstrated; they are required to maintain the *Acanthamoeba* life cycle [20], [23], [38]. Serine protease, the most abundant *Acanthamoeba* ES protein, is crucial for the encystment and excystment of *Acanthamoeba* [23]. Subtilisin and elastase, which are asthma inducers, have also been identified as *Acanthamoeba* ES proteins [39], [40]. Subtilisin has been isolated from several other organisms, including *Curvularia lunata*, *Bacillus subtilis*, *Epicoccum purpurascens*, and *Penicillium citrinum. S*ubtilisin elicits specific antibody production in mice and produce immediate and late onset allergic reactions in allergy patients [41]–[43]. In this study, using gelatin zymogram analysis, we also found that the major protease activity originated from serine protease (Figure 2). Inhibition of serine protease activity by specific inhibitors suppressed the expression of most chemokine genes with Th2 promoters, including *eotaxin*, *MDC*, and *TARC*, in lung epithelial cells (Figure 2). Our results show that ES proteins include several strong protease allergens that could elicit a Th2 response.

The induction of allergic airway inflammation by protease allergens occurs via several known mechanisms. In one mechanism, a protease allergen can activate DCs and induce IL-25, TSLP, and allergy-related chemokines [44]. In this study, *Acanthamoeba* ES proteins induced the activation of DCs, which accelerated the differentiation of naive CD4^+^ T cell into Th2 cells (Figure 3 & 4). Th2 cells are critical for the induction of allergic asthma via production of IL-4, IL-5, IL-13, and eotaxin [45], [46]. In addition, Th2 cells express CCR3, CCR4, and CCR8. *In vitro* studies have shown that MDC and TARC, high-affinity CCR4 ligands, induce the selective migration of Th2 cells [47] and IL-25-mediated Th2 responses in eosinophilia [45]. The results of this study show that *Acanthamoeba* contains ES proteins with serine protease activity that stimulate DCs and thereby promote the differentiation of naive T cells into Th2 cells (Figure 2, 3 & 4).

A second mechanism for the induction of allergic symptoms by protease allergens involves the digestion of cell surface molecules and tissue destruction. Der P1, a cysteine protease from the house dust mite, can cleave human cell surface molecules, including the low-affinity IgE receptor (CD23/FcεRII), the alpha-subunit of the IL-2 receptor (CD25), and the protease inhibitor alpha1-antitrypsin [48]. Proteolytic degradation of tight junctions in lung epithelium and the release of proinflammatory cytokines from bronchial epithelial cells, mast cells, and basophils in response to Der P1 have been reported [49]. In addition, the fungus *Alternaria alternata* is associated with asthma; its serine protease activity can disrupt the lung epithelial barrier [50]. However, we do not know whether *Acanthamoeba* ES proteins can cleave human cell surface molecules and/or cause tissue destruction in the manner of Der P1. Thus, further investigation is required to elucidate the exact mechanisms by which ES proteins induce the allergic response.

In a third mechanism for the induction of allergic symptoms by protease allergens, some proteases signal through PAR2 [3], [4], [11], [14], [51]. The effects of PAR2 activation on leukocyte motility, cytokine production, adhesion molecule expression, and a variety of other physiological or pathophysiological functions *in vitro* and *in vivo* have been described [4], [51], [52]. In *in vivo* experiments using PAR2 KO mice, we demonstrated that *Acanthamoeba* ES proteins elicited allergic airway inflammation via PAR2 (Figure 5). Production of Th2 cytokines in the spleen and LLNs and the eosinophil infiltration rate in the lung were significantly lower in ES protein-treated PAR2 KO mice than in ES protein-treated WT mice (Figure 5). In order to know how ES proteins interact with PAR2 molecule indirect/direct and what is key molecules in ES proteins on PAR2 activation, we need more detailed information from further studies. Although most clinical indices of allergic airway inflammation were reduced in PAR2 KO, few reached the basal level. In the present study, ES proteins upregulated *PAR2* expression 8-fold over control levels. They also activated *PAR3* and *PAR4* gene expression in lung epithelial cells (Figure 2C). Thus, although signaling via PAR2 was blocked in PAR2 KO mice, ES proteins might have activated the Th2 response via PAR3, PAR4, or other receptors.

Finally, *Acanthamoeba* and ES proteins significantly induced also neutrophilic inflammation in airway, it has the possibility related with Th17 cell mediate steroid resistant airway inflammation. However, Th17 response in *Acanthamoeba* and ES treatment was not significantly increased and we have little information about therapeutic effects of steroids on *Acanthamoeba* induced asthma model. The detailed mechanisms of neutrophilic inflammation by *Acanthamoeba* airway inflammation model will be reviled in next studies.

The KA/WP6 *Acanthamoeba* strain used in this study was isolated from domestic tap water. Genetically similar strains have been isolated from contact lens storage cases, swimming pools, air conditioning systems, and the corneas of amoebic keratitis patients [26]. We also detected *Acanthamoeba* antigens in house dust (Figure 6B), indicating that *Acanthamoeba* can be encountered in domestic environments, similar to house dust mite allergens. Therefore, it is not surprising that most healthy individuals possess specific IgG against *Acanthamoeba* [53]. However, to our knowledge, the levels of anti-*Acanthamoeba* IgE in human serum have not been previously reported. Of particular interest, we observed significantly higher *Acanthamoeba*-specific IgE levels in asthma patients than in healthy controls (Figure 6A). These results indicate that *Acanthamoeba* might be a novel human allergen.

In summary, we found that *Acanthamoeba* trophozoites and ES proteins elicited asthma-like symptoms and promoted Th2 responses by activating DCs via PAR2 signaling in a mouse model. In addition, we observed that asthma patients had higher *Acanthamoeba*-specific IgE titers than healthy controls. In conclusion, we suggest that future studies attempt to identify specific major allergens among the ES proteins of *Acanthamoeba*, which could aid in the diagnosis of allergic airway conditions.

## Supporting Information

Figure S1 **High-dose** ***Acanthamoeba*** **trophozoite infection induces severe allergic airway inflammation.** (A) Tissue inflammation observed on stained lung sections (a and c: PBS-treated; b and d: *Acanthamoeba*-infected; a and b, H&E-stained; c and d, PAS-stained). (B) Cytokine concentrations in BALF and in the culture medium of CD3-stimulated lymphocytes isolated from LLNs were measured. (*p<0.05, ** <0.01, ***p<0.001).
(PPT)

Figure S2 **Eosinophils are recruited to the airway by low-dose** ***Acanthamoeba*** **infection.** (A) Airway resistance values in response to methacholine (0 to 50 mg/ml). (B) Differential cell count in 800 μl BAL after Diff-Quik staining. (C) Total and *Acanthamoeba*-specific IgE levels were measured in serum by ELISA. (*p<0.05, **p<0.01, ***p<0.001).
(PPT)
