# Supplementary material for: Acanthamoeba Protease Activity Promotes Allergic Airway Inflammation via Protease-Activated Receptor 2
Source: PLoS One. 2014 Mar 21;9(3):e92726. doi: 10.1371/journal.pone.0092726 (PMC3962434; doi:10.1371/journal.pone.0092726)
Supplement: Figure S1 — High-dose Acanthamoeba trophozoite infection induces severe allergic airway inflammation. (A) Tissue inflammation observed on stained lung sections (a and c: PBS-treated; b and d: Acanthamoeba-infected; a and b, H&E-stained; c and d, PAS-stained). (B) Cytokine concentrations in BALF and in the culture medium of CD3-stimulated lymphocytes isolated from LLNs were measured. (*p<0.05, ** <0.01, ***p<0.001). (PPT) [file pone.0092726.s001.ppt]

## Slide 1
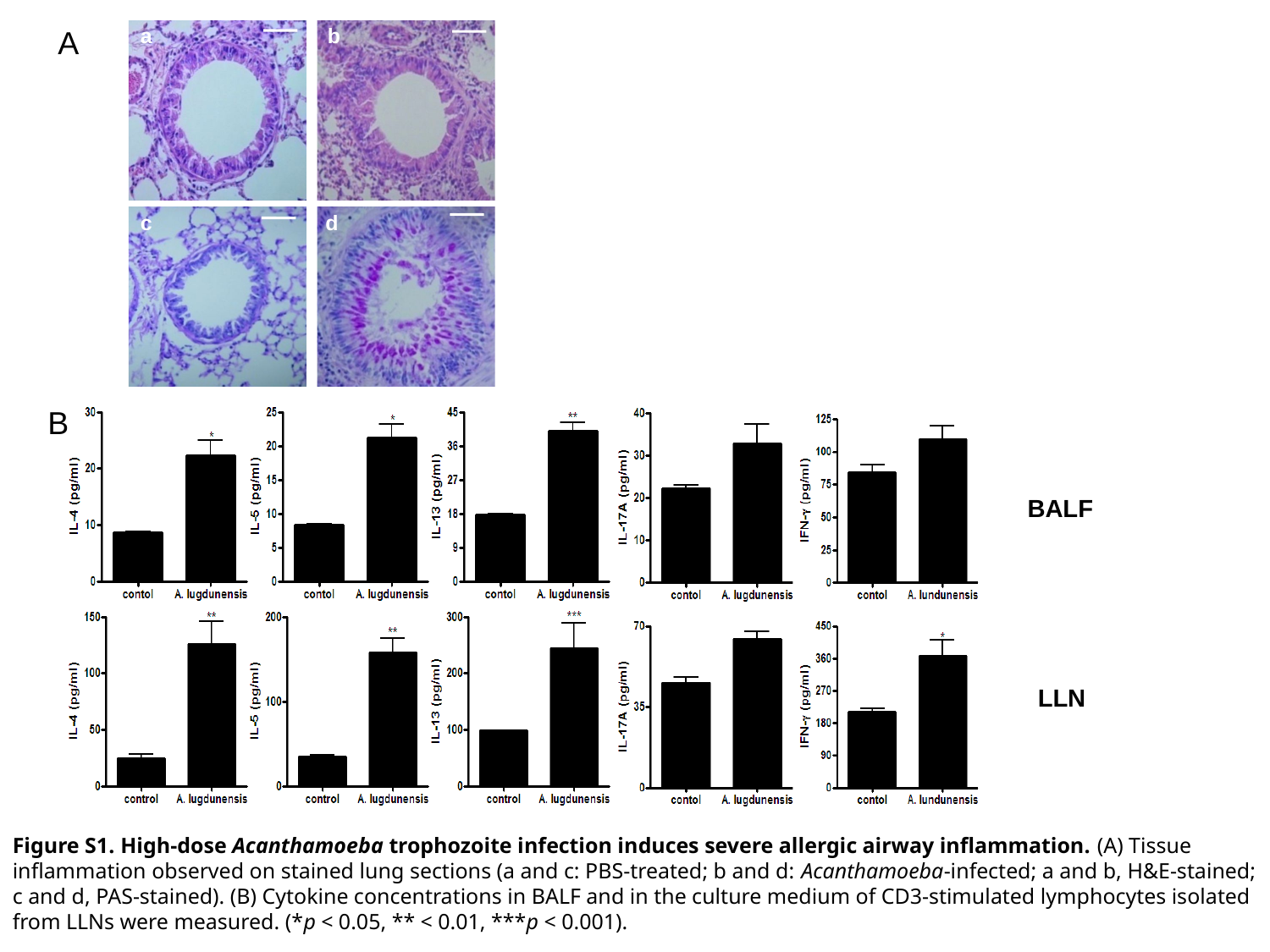

A
a
b
c
d
B
BALF
LLN
Figure S1. High-dose Acanthamoeba trophozoite infection induces severe allergic airway inflammation. (A) Tissue inflammation observed on stained lung sections (a and c: PBS-treated; b and d: Acanthamoeba-infected; a and b, H&E-stained; c and d, PAS-stained). (B) Cytokine concentrations in BALF and in the culture medium of CD3-stimulated lymphocytes isolated from LLNs were measured. (*p < 0.05, ** < 0.01, ***p < 0.001).
