# Supplementary material for: Acanthamoeba Protease Activity Promotes Allergic Airway Inflammation via Protease-Activated Receptor 2
Source: PLoS One. 2014 Mar 21;9(3):e92726. doi: 10.1371/journal.pone.0092726 (PMC3962434; doi:10.1371/journal.pone.0092726)
Supplement: Figure S2 — Eosinophils are recruited to the airway by low-dose Acanthamoeba infection. (A) Airway resistance values in response to methacholine (0 to 50 mg/ml). (B) Differential cell count in 800 μl BAL after Diff-Quik staining. (C) Total and Acanthamoeba-specific IgE levels were measured in serum by ELISA. (*p<0.05, **p<0.01, ***p<0.001). (PPT) [file pone.0092726.s002.ppt]

## Slide 1
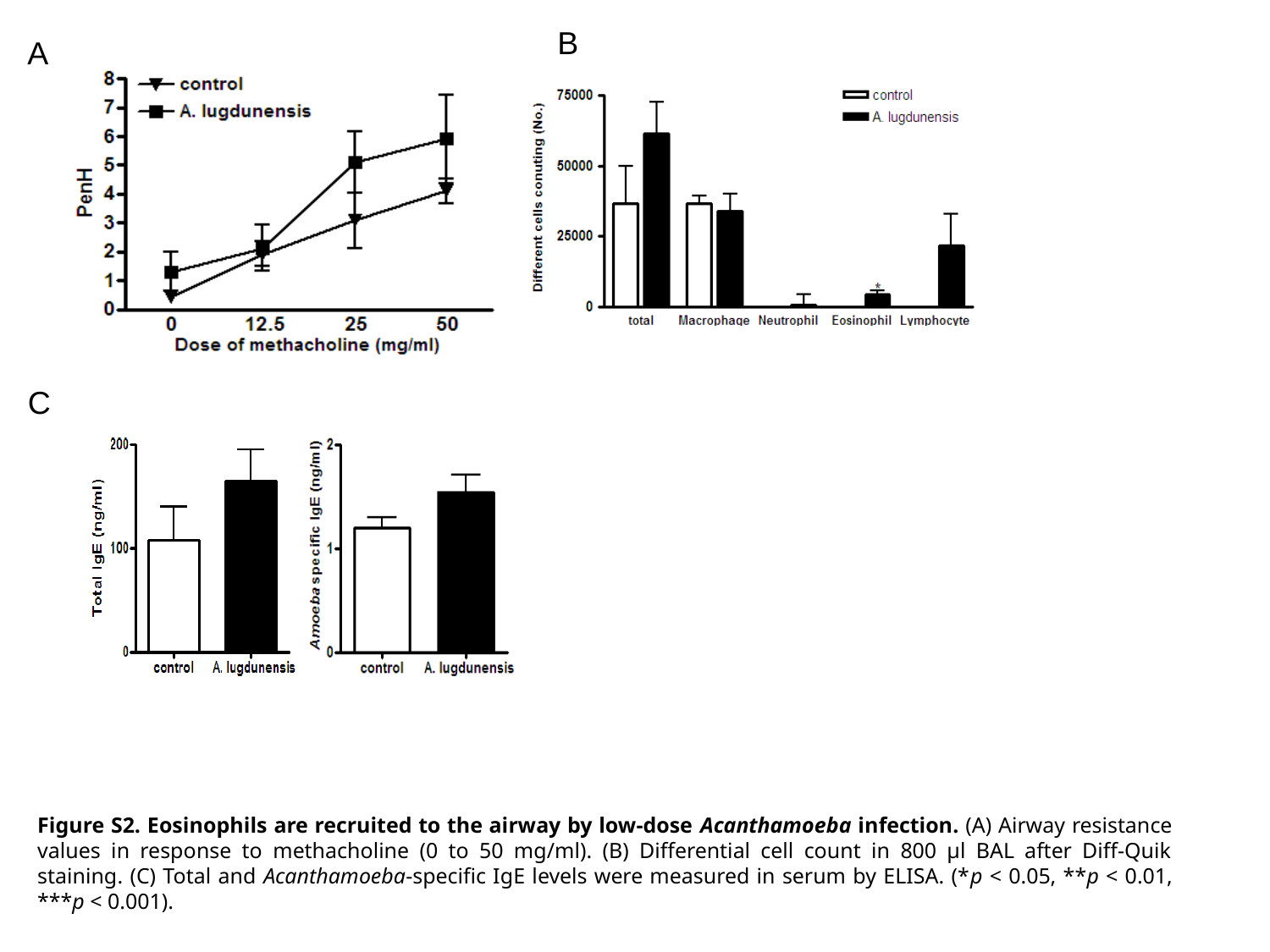

B
A
C
Figure S2. Eosinophils are recruited to the airway by low-dose Acanthamoeba infection. (A) Airway resistance values in response to methacholine (0 to 50 mg/ml). (B) Differential cell count in 800 µl BAL after Diff-Quik staining. (C) Total and Acanthamoeba-specific IgE levels were measured in serum by ELISA. (*p < 0.05, **p < 0.01, ***p < 0.001).
